# RIOK3 sustains colorectal cancer cell survival under glucose deprivation via an HSP90α-dependent pathway

**DOI:** 10.1038/s41389-024-00514-5

**Published:** 2024-03-07

**Authors:** Nan Zhang, Lu Dong, Tingting Ning, Feng Du, Mengran Zhao, Junxuan Xu, Sian Xie, Si Liu, Xiujing Sun, Peng Li, Shutian Zhang, Shengtao Zhu

**Affiliations:** 1 Department of Gastroenterology, Beijing Friendship Hospital, Capital Medical University, National Clinical Research Center for Digestive Disease, Beijing Digestive Disease Center, Beijing Key Laboratory for Precancerous Lesion of Digestive Disease, Beijing, China; 2 Affiliated Hospital of Weifang Medical University, School of Clinical Medicine, Weifang Medical University, Weifang, China

**Keywords:** Cancer metabolism, Cell death

## Abstract

Glucose oxidation via the pentose phosphate pathway serves as the primary cellular mechanism for generating nicotinamide adenine dinucleotide phosphate (NADPH). The central regions of solid tumors typically experience glucose deficiency, emphasizing the need for sustained NADPH production crucial to tumor cell survival. This study highlights the crucial role of RIOK3 in maintaining NADPH production and colorectal cancer (CRC) cell survival during glucose deficiency. Our findings revealed upregulated RIOK3 expression upon glucose deprivation, with RIOK3 knockout significantly reducing cancer cell survival. Mechanistically, RIOK3 interacts with heat shock protein 90α (HSP90α), a chaperone integral to various cellular processes, thereby facilitating HSP90α binding to isocitrate dehydrogenase 1 (IDH1). This interaction further upregulates IDH1 expression, enhancing NADPH production and preserving redox balance. Furthermore, RIOK3 inhibition had no discernible effect on intracellular NADPH levels and cell death rates in HSP90α-knockdown cells. Collectively, our findings suggest that RIOK3 sustains colon cancer cell survival in low-glucose environments through an HSP90α-dependent pathway. This highlights the significance of the RIOK3–HSP90α–IDH1 cascade, providing insights into potential targeted therapeutic strategies for CRC in metabolic stress conditions.

## Introduction

Cancer cell survival under metabolic stress represents a crucial stage for both solid tumor growth and metastatic progression. Exploring the mechanisms governing their survival is crucial in the realm of cancer therapy [1, 2]. Glucose, a crucial energy substrate for cancer, plays a central role in cancer cell metabolism. Glucose deprivation represents a major form of metabolic stress in cancer cells [3]. Glucose starvation impairs glycolysis and the pentose phosphate pathway, leading to oxidative stress attributed to the increased production of reactive oxygen species (ROS) and a weakened antioxidant system. This results in redox imbalance and eventual cell death [4].

RIOK3, a member of the right open reading frame (RIO) kinase family, is an evolutionarily conserved atypical serine/threonine protein kinase in eukaryotes [5]. RIOK3 functions as an oncogene in pancreatic cancer and glioma, promoting tumor proliferation and migration [6–8]. Furthermore, RIOK3 plays a crucial role in maintaining the cytoskeletal organization of actin filaments required for migration and invasion in hypoxia-driven tumor metastasis [9]. However, the impact of RIOK3 on the survival of tumor cells in a glucose-deprived environment remains unclear.

Heat shock protein 90 (HSP90) is a pivotal molecular chaperone integral to cellular regulation, widely recognized for its vital role in fostering cancer cell proliferation [10]. Through interactions with various cancer-associated proteins, HSP90 ensures the accurate folding and maintenance of their active states [11]. Furthermore, HSP90 exerts a critical protective function during cellular stress, assisting cells in adapting to challenging environments by stabilizing their interacting protein partners [12].

In this study, we aimed to investigate the role of increased RIOK3 expression in regulating tumor cell survival under low glucose levels in CRC. We monitored the interactions between RIOK3 and HSP90α, as well as its impact on the association of HSP90α with its substrate isocitrate dehydrogenase 1 (IDH1), an enzyme catalyzing the final step in the transfer of hydride ions to NADP^+^ [13]. This, in turn, enhances NADPH generation, leading to the clearance of ROS and the maintenance of the oxidative-reductive balance in tumor cells. We believe that targeting the RIOK3-HSP90α-IDH1 cascade reaction under glucose starvation conditions could be a promising metabolic blocker target for antitumor therapy.

## Results

### Glucose depletion induces RIOK3 expression in CRC cells

**Fig. 1:**
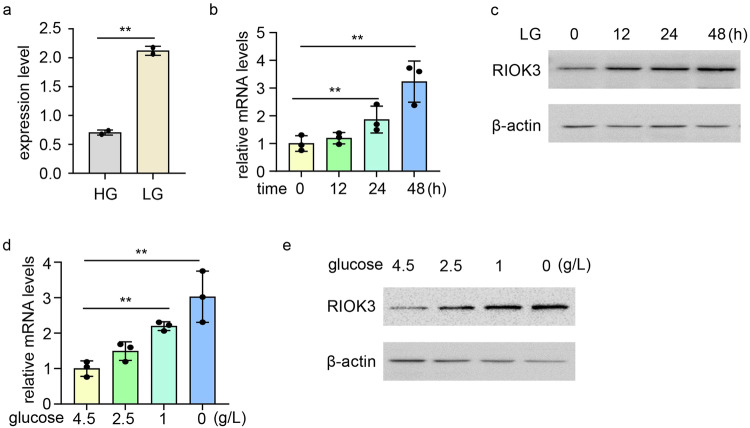
RIOK3 expression is increased under glucose deficiency. **a** RIOK3 mRNA levels increase under low-glucose conditions based on GEO datasets (GSE31084). **b** HCT116 cells were incubated in low-glucose DMEM for the indicated times. RNA was then extracted and RIOK3 mRNA levels were analyzed with qPCR. Data are shown as mean ± SD (*n* = 3). **c** HCT116 cells were incubated in low-glucose DMEM for the indicated times. Western blotting was performed to detect RIOK3 protein levels. **d** HCT116 cells were cultured in a medium with a specific glucose concentration for 24 h. RNA was then extracted and RIOK3 mRNA levels were analyzed with qPCR. Data are shown as mean ± SD (*n* = 3). **e** HCT116 cells were cultured in a medium with a specific glucose concentration for 24 h. Western blotting was performed to detect RIOK3 protein levels.

To elucidate the molecular mechanisms guiding the adaptive response of colon cancer cells to glucose deprivation, we monitored the gene expression profiles of HCT116 human CRC cells under high- and low-glucose conditions (GSE31084). A substantial elevation in RIOK3 expression was observed under low-glucose conditions compared with that under high-glucose medium (Fig. 1a).

To validate the impact of glucose deprivation on RIOK3 expression, HCT116 and SW480 colon cancer cells were exposed to a low-glucose (1 g/L) medium. An evident time-dependent increase in RIOK3 mRNA was noted in HCT116 (Fig. 1b). RIOK3 protein levels were also increased in low-glucose medium (Fig. 1c). Consistent results were obtained in SW480 cells, where low-glucose treatment resulted in elevated RIOK3 mRNA and protein levels (Supplementary Fig. S1a, b). We also examined RIOK3 expression in HCT116 cells across varying glucose concentrations, revealing a direct correlation between decreased glucose levels and increased RIOK3 expression in colon cancer cells (Fig. 1d, e).

Additionally, we assessed RIOK3 expression in the normal human colon cell line NCM460 and FHC. As shown in Supplementary Fig. S1c, d, we did not observe significant upregulation of RIOK3 expression upon glucose starvation in either NCM460 or FHC. This suggests that normal cells may employ a unique pathway, distinct from cancer cells, to adapt to glucose starvation.

### RIOK3 facilitates cell survival upon glucose deprivation

Given its established role in cancer proliferation and metastasis [6, 7], we investigated whether RIOK3 abundance is imperative for CRC cell survival under glucose limitation.

**Fig. 2:**
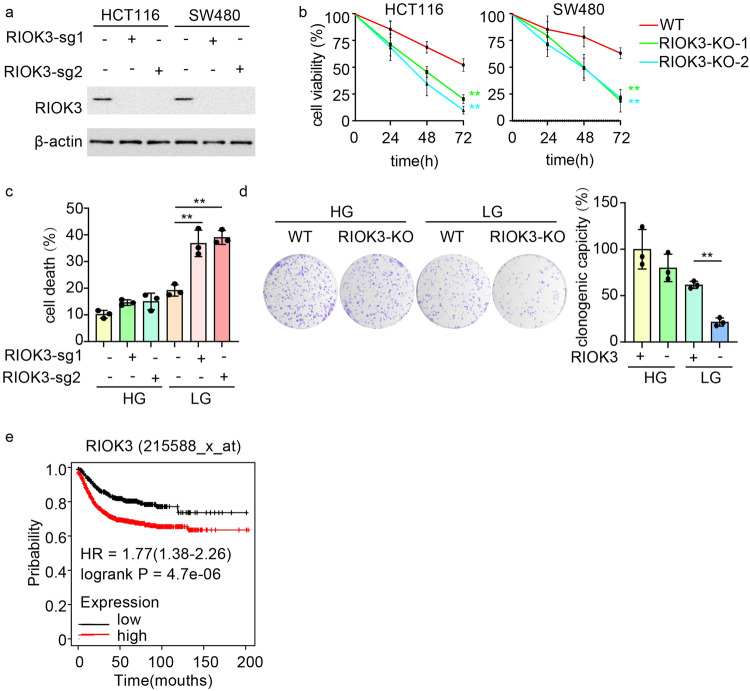
RIOK3 supports cell survival under glucose starvation condition. **a** Western blotting was performed to determine RIOK3 protein levels. **b** WT and RIOK3-KO CRC cells were cultured in low-glucose DMEM for the indicated times. Cell viability was then tested with the CCK-8 assay. Data are shown as mean ± SD (*n* = 3). **c** WT and RIOK3-KO HCT116 cells were cultured in low-glucose DMEM for 48 h, and the cell death rate was measured by 7-AAD staining assay. Data are shown as mean ± SD (*n* = 3). **d** WT and RIOK3-KO HCT116 cells were cultured in high-glucose or low-glucose DMEM, and a clonogenic survival assay was performed. Colonies were counted after crystal violet staining. Right graph, the quantitative data are shown as the mean ± SD (*n*= 3). **e** Kaplan–Meier plots were generated to compare the overall survival between the high-expression and low-expression groups of RIOK3.

To this end, we generated RIOK3-KO cell lines using the CRISPR-Cas9 system (Fig. 2a). CCK-8 assay results indicated a significantly higher viability of wild-type (WT) cells compared with that of RIOK3-KO cells under low-glucose conditions (Fig. 2b). We also analyzed the cell death rate under low-glucose conditions and the ability of surviving cells to grow and form colonies. RIOK3-KO HCT116 cells exhibited increased sensitivity to glucose deprivation-induced cell death compared with that in control cells (Fig. 2c and Supplementary Fig. S2a). For the colony formation assay, WT and RIOK3-KO HCT116 cells were subjected to a period of starvation prior to subsequent cultivation in regular growth medium. RIOK3 silencing substantially impaired colony formation in seeded cells under starvation (Fig. 2d), supporting the notion that RIOK3 plays a role in promoting tumor growth and survival, particularly under nutrient-deprived conditions.

Furthermore, a prognostic analysis of RIOK3 in colon cancer using the Kaplan –Meier plotter database tool revealed that reduced overall survival in colon cancer patients was linked to the elevated expression level of RIOK3 (Fig. 2e), highlighting the significant role of RIOK3 in CRC progression.

### RIOK3 knockout induces ROS generation-triggered cell death

**Fig. 3:**
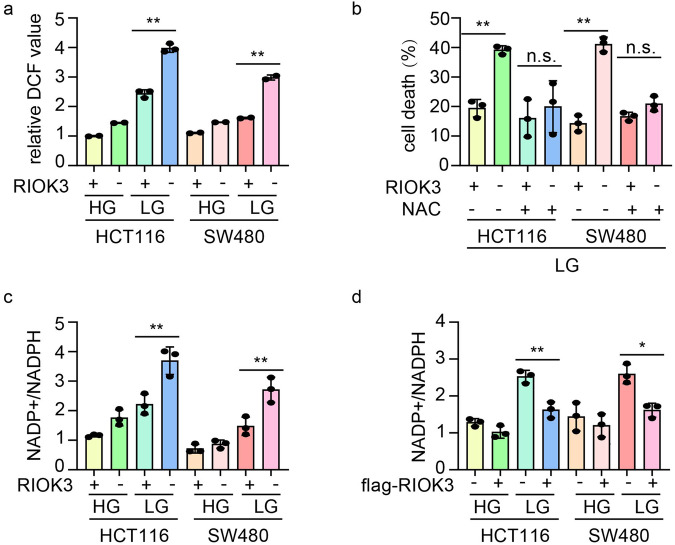
Altered RIOK3 expression affects ROS. **a** WT and RIOK3-KO CRC cells were cultured in low-glucose DMEM for 48 h, and the ROS levels were quantified as the mean DCF values. Data are shown as mean ± SD (*n* = 3). **b** WT and RIOK3-KO CRC cells were cultured in low-glucose DMEM in the presence or absence of 10 mM NAC for 48 h, and the cell death rate was measured by 7-AAD staining assay. Data are shown as mean ± SD (*n* = 3). **c** WT and RIOK3-KO CRC cells were cultured in low-glucose DMEM for 48 h, and the NADP^+^/NADPH ratio was determined. Data are shown as mean ± SD (*n* = 3). **d** CRC cells were transfected with an empty vector or flag-RIOK3 plasmids. 24 h after transfection, cells were cultured in low-glucose DMEM for 48 h, and the NADP^+^/NADPH ratio was determined. Data are shown as mean ± SD (*n* = 3).

Oxidative stress is a primary trigger for cell death under nutrient deficiency [14, 15]. Accordingly, we monitored ROS production following RIOK3 knockout using DCFH-DA fluorescence. Loss of RIOK3 significantly elevated ROS levels in CRC cells, particularly under glucose-deprived conditions (Fig. 3a). Intracellular ROS levels were also assessed at different time points under low glucose conditions, revealing a gradual increase with prolonged exposure to low glucose, closely correlated with RIOK3 expression (Supplementary Fig. S3a). To confirm whether the impact of RIOK3 on cell survival under low-glucose conditions was associated with ROS generation, cells were treated with the ROS scavenger N-acetyl-l-cysteine (NAC), which resulted in the reversal of cell death in RIOK3-KO cells under starvation (Fig. 3b and Supplementary Fig. S3b, c).

NADPH is a key factor in maintaining intracellular redox homeostasis [16]. Therefore, we examined the impact of RIOK3 on intracellular NADPH levels. Indeed, the loss of RIOK3 led to a reduction in NADPH levels, whereas the gain of RIOK3 function consistently elevated them (Fig. 3c, d). This suggests that RIOK3 regulates NADPH homeostasis, thereby enhancing cancer cell survival against oxidative stress.

### RIOK3 interacts with HSP90α and subsequently augments IDH1 expression

**Fig. 4:**
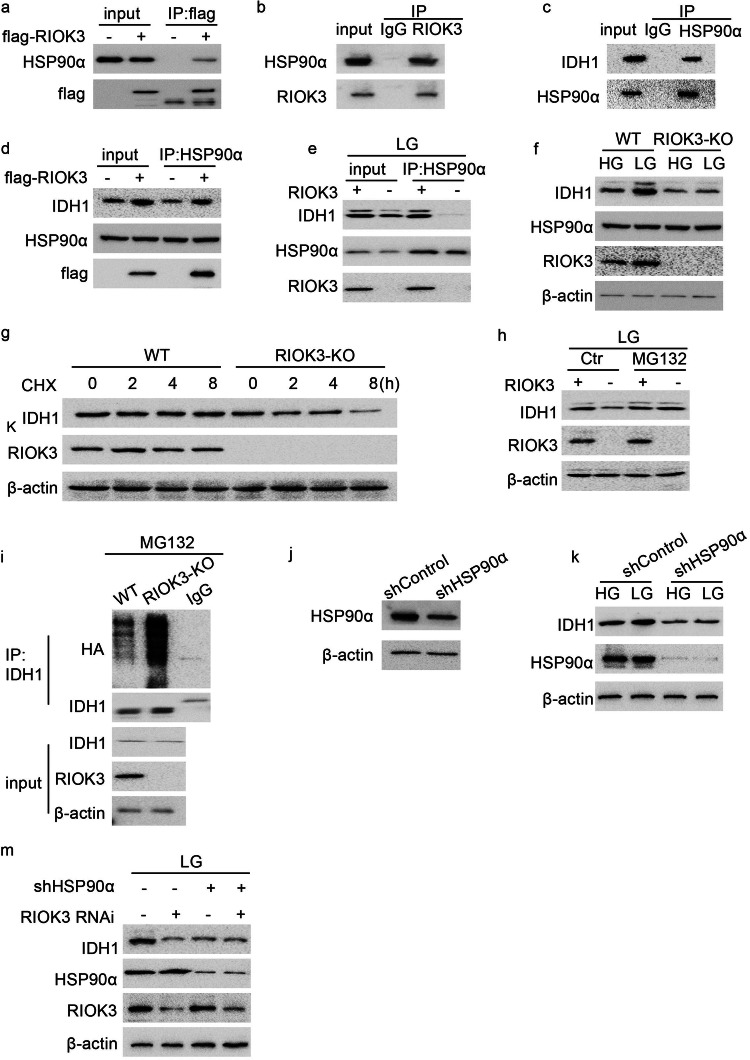
High expression of RIOK3 promotes the binding between HSP90α and IDH1. **a** HCT116 cells were transfected with an empty vector or flag-RIOK3 plasmids. 24 h after transfection, the cell lysate was extracted for co-IP with anti-flag antibody. **b** HCT116 cell lysates were extracted for co-IP using anti-RIOK3 antibody. **c** HCT116 cell lysates were extracted for co-IP using anti-HSP90α antibody. **d** HCT116 cells were transfected with an empty vector or flag-RIOK3 plasmids. 24 h after transfection, the cell lysate was extracted for co-IP with anti-HSP90α antibody. **e** WT and RIOK3-KO HCT116 cells were cultured in low-glucose DMEM for 48 h, and the cell lysate was extracted for co-IP with anti-HSP90α antibody. **f** WT and RIOK3-KO HCT116 cells were cultured in low-glucose DMEM for 48 h, western blotting was performed to determine IDH1, HSP90α, and RIOK3 protein levels. **g** WT and RIOK3-KO HCT116 cells were treated with or without 50 μM CHX, and western blotting was used to detect IDH1 level. **h** WT and RIOK3-KO HCT116 cells were cultured in low-glucose DMEM for 48 h and treated with or without 2 μM MG132, and western blotting was used to detect IDH1 level. **i**. WT and RIOK3-KO HCT116 cells expressing HA-Ubiquitin plasmids were cultured in low-glucose DMEM for 48 h with 2 μM MG132, and the cell lysate was extracted for co-IP with anti-IDH1 antibody. **j** Western blotting was performed to determine HSP90α protein levels. **k** HCT116 cells expressing control-shRNA or HSP90α-shRNA were cultured in low-glucose DMEM for 48 h, and western blotting was performed to determine IDH1 and HSP90α protein levels. **m** HCT116 cells expressing control-shRNA or HSP90α-shRNA were transfected with a non-specific siRNA or RIOK3 siRNA. 24 h after transfection, cells were cultured in low-glucose DMEM for 48 h. Western blotting was performed to determine IDH1, HSP90α, and RIOK3 protein levels.

To explore the mechanism by which RIOK3 regulates intracellular NADPH levels, we conducted mass spectrometry (MS) analysis to identify its binding partners. MS results revealed an interaction between RIOK3 and HSP90α, a molecular chaperone crucial for various oncogenic processes [17]. Subsequent co-immunoprecipitation (co-IP) experiments were performed to validate the interaction between RIOK3 and HSP90α. Lysates of HCT116 cells expressing flag-RIOK3 were immunoprecipitated with an anti-flag antibody. The immunoprecipitated complex was then analyzed using western blotting with an antibody against HSP90α. As shown in Fig. 4a, these analyses confirmed the physical association between flag-RIOK3 and HSP90α. Employing the RIOK3 antibody for the co-IP assays similarly revealed an observed interaction between RIOK3 and HSP90α (Fig. 4b), providing further evidence of their interplay in the cellular context.

The NADPH-producing enzyme IDH1 is reportedly an interacting partner of HSP90 [18]. Therefore, we validated the interaction between HSP90α and IDH1 in colon cancer cells. The co-IP experiment demonstrated the binding of HSP90α to IDH1 (Fig. 4c). Notably, RIOK3 overexpression enhanced both IDH1 protein levels and its interaction with HSP90α (Fig. 4d). Subsequently, we examined the binding between HSP90α and IDH1 in RIOK3-KO HCT116 cells. As can be seen in Fig. 4e, an interaction between IDH1 and HSP90α was observed in a low-glucose environment. However, in RIOK3-KO cells, both the expression of IDH1 and its binding with HSP90α were substantially decreased. Therefore, we hypothesized that RIOK3 modulates IDH1 expression by influencing its interaction with HSP90α in response to glucose availability.

Concurrently evaluating the influence of different glucose concentrations on IDH1 expression, we observed an increase in IDH1 expression as glucose concentration decreased, consistent with RIOK3 (Supplementary Fig. S4a). To further validate this regulatory role of RIOK3, we treated the RIOK3-KO cell line with low-glucose culture medium and confirmed that low glucose conditions failed to induce the expression of IDH1 in RIOK3-KO cells (Fig. 4f). We hypothesized that RIOK3 might influence the expression of IDH1 by affecting its stability. To validate this hypothesis, we conducted a cycloheximide (CHX) chase experiment and observed a significantly accelerated degradation rate of IDH1 in RIOK3-KO cells (Fig. 4g), indicating that RIOK3 exerts an impact on IDH1 expression by affecting its stability. To further clarify the mechanism through which RIOK3 regulates IDH1 stability and considering potential ubiquitination sites on IDH1 [19], we treated HCT116 cells with MG132, a cell-permeable proteasome inhibitor, to determine whether IDH1 could undergo ubiquitination degradation. As illustrated in Fig. 4h, MG132 restored reduced IDH1 levels caused by RIOK3-KO under low-glucose conditions. Subsequently, we transfected HA-ubiquitin plasmids into both WT and RIOK3-KO HCT116 cells and observed increased ubiquitination of IDH1 in RIOK3-KO cells (Fig. 4i). This indicates that RIOK3 enhances IDH1 stability by reducing its ubiquitination levels.

To further investigate the role of HSP90α, HSP90α-knockdown (KD) HCT116 cell lines were generated using shRNA (Fig. 4j and Supplementary Fig. S4b). Intriguingly, we observed a decrease in IDH1 expression in HSP90α-KD cells (Fig. 4k), yet its expression was unaffected by RIOK3 (Fig. 4m). We further validated the impact of HSP90 activity on IDH1 expression using the HSP90 inhibitor 17-AAG and observed that 17-AAG treatment suppressed IDH1 expression, and low glucose did not lead to a significant increase in IDH1 expression (Supplementary Fig. S4c). Furthermore, 17-AAG treatment attenuated the differences in IDH1 expression between WT and RIOK3-KO cells (Supplementary Fig. S4d), further confirming that RIOK3 modulates IDH1 expression through HSP90α.

### RIOK3 regulates NADPH production and maintains redox balance through HSP90α

**Fig. 5:**
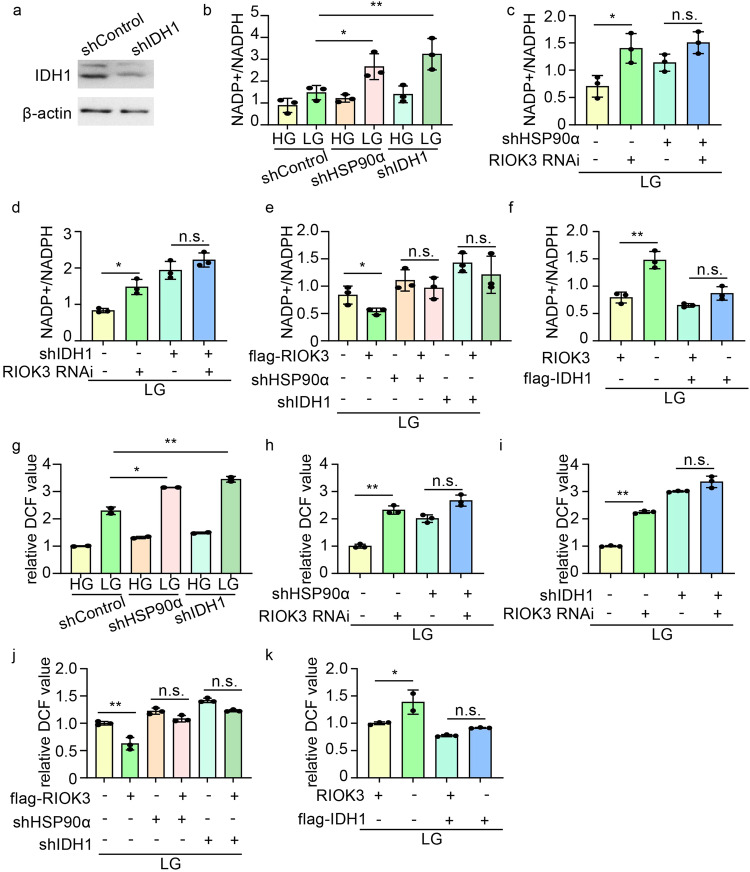
RIOK3 modulates NADPH generation and preserves redox equilibrium via HSP90α. **a** Western blotting was performed to determine IDH1 protein levels. **b** HCT116 cells expressing control-shRNA, HSP90α-shRNA, or IDH1-shRNA were cultured in low-glucose DMEM for 48 h, and NADP^+^/NADPH ratio was determined. Data are shown as mean ± SD (*n* = 3). HCT116 cells expressing control-shRNA, HSP90α-shRNA (**c**), or IDH1-shRNA (**d**) were transfected with a non-specific siRNA or RIOK3 siRNA. 24 h after transfection, cells were cultured in low-glucose DMEM for 48 h. The NADP^+^/NADPH ratio was then determined. Data are shown as mean ± SD (*n* = 3). **e** HCT116 cells expressing control-shRNA, HSP90α-shRNA, or IDH1-shRNA were transfected with an empty vector or flag-RIOK3 plasmids. 24 h after transfection, cells were cultured in low-glucose DMEM for 48 h. The NADP^+^/NADPH ratio was then determined. Data are shown as mean ± SD (*n* = 3). **f** WT and RIOK3-KO HCT116 cells were transfected with an empty vector or flag-IDH1 plasmids, 24 h after transfection, and cells were cultured in low-glucose DMEM for 48 h. The NADP^+^/NADPH ratio was then determined. Data are shown as mean ± SD (*n* = 3). **g** HCT116 cells expressing control-shRNA, HSP90α-shRNA, or IDH1-shRNA were cultured in low-glucose DMEM for 48 h, and the ROS levels were quantified as the mean DCF values. Data are shown as mean ± SD (*n* = 3). HCT116 cells expressing control-shRNA, HSP90α-shRNA (**h**), or IDH1-shRNA (**i**) were transfected with a non-specific siRNA or RIOK3 siRNA. 24 h after transfection, cells were cultured in low-glucose DMEM for 48 h. ROS levels were then quantified as the mean DCF values. Data are shown as mean ± SD (*n* = 3). **j** HCT116 cells expressing control-shRNA, HSP90α-shRNA, or IDH1-shRNA were transfected with an empty vector or flag-RIOK3 plasmids. 24 h after transfection, cells were cultured in low-glucose DMEM for 48 h. ROS levels were then quantified as the mean DCF values. Data are shown as mean ± SD (*n* = 3). **k** WT and RIOK3-KO HCT116 cells were transfected with an empty vector or flag-IDH1 plasmids, 24 h after transfection, and cells were cultured in low-glucose DMEM for 48 h. ROS levels were then quantified as the mean DCF values. Data are shown as mean ± SD (*n* = 3).

To further elucidate whether the impact of RIOK3 on NADPH production is linked to its binding to HSP90α, we generated IDH1-KD HCT116 cell lines using shRNA (Fig. 5a and Supplementary Fig. S5a). Knocking down either HSP90α or IDH1 decreased NADPH levels in colon cancer cells, especially under low-glucose conditions (Fig. 5b). RIOK3 inhibition did not significantly affect NADPH levels in HSP90α-KD colon cancer cells compared with that in the control cells (Fig. 5c), suggesting that HSP90α plays a crucial role in mediating RIOK3’s impact on NADPH metabolism. Concordantly, the utilization of 17-AAG similarly attenuated the impact of RIOK3 on NADPH (Supplementary Fig. S5b). Similarly, RIOK3 inhibition failed to induce a substantial disparity in NADPH levels in IDH1-KD CRC cells compared with that in the control group (Fig. 5d). Subsequently, we overexpressed flag-RIOK3 in HSP90α-KD and IDH1-KD cells and observed that overexpression of RIOK3 in low-glucose environments led to increased NADPH levels, and this effect was attenuated when HSP90α or IDH1 was knocked down (Fig. 5e). Furthermore, IDH1 overexpression in RIOK3-KO cells partially restored the inhibitory effect of RIOK3 on NADPH (Fig. 5f), further supporting the notion that the regulatory effect of RIOK3 on NADPH production likely depends on HSP90α and IDH1.

We also observed that depletion of either HSP90α or IDH1 led to differential ROS elevation in HSP90α-KD and IDH1-KD cells, respectively (Fig. 5g). Notably, RIOK3 inhibition did significantly alter the ROS levels in these cells compared with that in control cells (Fig. 5h, i). We observed comparable ROS levels in WT and RIOK3-KO cells post-17-AAG treatment (Supplementary Fig. S5c). The influence of RIOK3 overexpression on ROS production was mitigated by HSP90α or IDH1 knockdown (Fig. 5j). Notably, IDH1 overexpression in RIOK3-KO cells partially reversed RIOK3’s inhibitory influence on ROS (Fig. 5k).

Collectively, these findings imply that RIOK3 plays a pivotal role in mediating the maintenance of redox balance and regulation of ROS levels in colon cancer cells through HSP90α- and IDH1-dependent mechanisms.

### RIOK3 supports colon cancer cell survival under glucose starvation via HSP90α

**Fig. 6:**
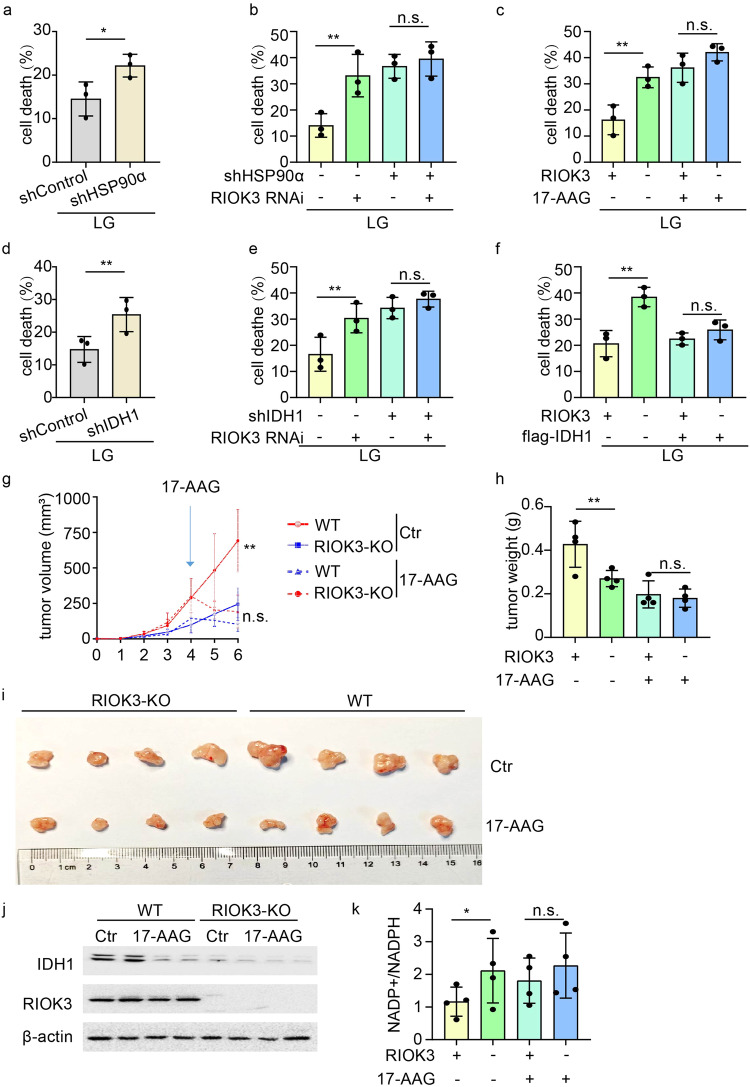
RIOK3‐promoted cell survival under glucose starvation is mediated via HSP90α. **a** HCT116 cells expressing control-shRNA or HSP90α-shRNA were cultured in low-glucose DMEM for 48 h, and the cell death rate was measured by 7-AAD staining assay. Data are shown as mean ± SD (*n* = 3). **b** HCT116 cells expressing control-shRNA or HSP90α-shRNA were transfected with a non-specific siRNA or RIOK3 siRNA. 24 h after transfection, cells were cultured in low-glucose DMEM for 48 h, and the cell death rate was measured by 7-AAD staining assay. Data are shown as mean ± SD (*n* = 3). **c** WT and RIOK3-KO HCT116 cells were cultured in low-glucose DMEM with 400 nM 17-AAG for 48 h, and the cell death rate was measured by 7-AAD staining assay. Data are shown as mean ± SD (*n* = 3). **d** HCT116 cells expressing control-shRNA or IDH1-shRNA were cultured in low-glucose DMEM for 48 h, and the cell death rate was measured by 7-AAD staining assay. Data are shown as mean ± SD (*n* = 3). **e** HCT116 cells expressing control-shRNA or IDH1-shRNA were transfected with a non-specific siRNA or RIOK3 siRNA. 24 h after transfection, cells were cultured in low-glucose DMEM for 48 h, and the cell death rate was measured by 7-AAD staining assay. Data are shown as mean ± SD (*n* = 3). **f** WT and RIOK3-KO HCT116 cells were transfected with an empty vector or flag-IDH1 plasmids, 24 h after transfection, cells were cultured in low-glucose DMEM for 48 h, and the cell death rate was measured by 7-AAD staining assay. Data are shown as mean ± SD (*n* = 3). **g** WT and RIOK3-KO HCT116 cells were injected into BALB/c nude mice, and tumor growth was monitored (*n* = 4). **h**, **i** Xenograft weight and size were measured (*n* = 4). **j** Protein was extracted from the tumors and analyzed with western blotting. **k** The tumor tissues were harvested for NADP^+^/NADPH detection. Data are shown as mean ± SD (*n* = 4).

An increased cell death rate in HSP90α-KD cells under low-glucose conditions compared with that in the control group was observed (Fig. 6a and Supplementary Fig. S6a). Interestingly, RIOK3 knockdown did not significantly impact cell death rates in HSP90α-KD cells (Fig. 6b and Supplementary Fig. S6b). Likewise, treating RIOK3-KO cells with the HSP90 inhibitor 17-AAG diminished the differences in cell death rates (Fig. 6c and Supplementary Fig. S6c), suggesting that RIOK3 inhibition did not substantially impact cell death in the absence of HSP90. Similarly, cell death increased in IDH1-KD cells compared with that in the control cells (Fig. 6d and Supplementary Fig. S6d). However, we observed that cell death rates did not significantly differ in IDH1-KD cells with either negative control or RIOK3 knockdown, indicating that RIOK3 knockdown did not significantly affect cell death in the absence of IDH1 (Fig. 6e and Supplementary Fig. S6e). Furthermore, IDH1 overexpression in RIOK3-KO HCT116 cells partially counteracted the inhibitory effects of RIOK3 knockout on the viability of colon cancer cells (Fig. 6f and Supplementary Fig. S6f).

We validated the effect of RIOK3 on colon cancer development in vivo using a nude mouse tumorigenesis experiment. Loss of RIOK3 inhibits tumor growth, whereas intraperitoneal injection of HSP90 inhibitor 17-AAG diminished the disparity in tumor size between WT and RIOK3-KO cells (Fig. 6g–i)). IDH1 expression and intratumoral NADPH levels in subcutaneous tumors of nude mice were assessed simultaneously. Consistent with the findings from in vitro cell experiments, RIOK3-KO tumors exhibited reduced IDH1 expression (Fig. 6j), accompanied by a decrease in NADPH levels (Fig. 6k). Furthermore, immunohistochemistry experiments to assess RIOK3 and IDH1 expression in WT tumors (Supplementary Fig. S7a) revealed increased expression levels of both RIOK3 and IDH1 in the central region of the tumor, consistent with the presence of a glucose-deficient environment in this region (Supplementary Fig. S7b).

In summary, our findings revealed that the role of RIOK3 in enhancing the tolerance of colon cancer cells to glucose deprivation is contingent on HSP90α activity.

## Discussion

The tumor microenvironment is typically characterized by diminished glucose availability owing to rapid cell growth and inadequate blood circulation [20]. Insufficient glucose can impede cell growth by diminishing energy and metabolite levels [21]. Consequently, cancer cells undergo adaptive responses, including the activation of signaling pathways and metabolic reprogramming, to survive under such constrained conditions [22]. Investigating how tumors adapt to glucose-deficient environments is pivotal for comprehending tumor progression. Our study underscores RIOK3 as a crucial player conferring protection under low-glucose conditions, with its expression increasing under glucose deprivation and proving indispensable for CRC cell survival. While RIOK3 has been previously reported to be associated with actin cytoskeleton organization and metastasis in hypoxic cells [9], our findings highlight its critical function in low-glucose environments, where it maintains redox equilibrium.

Glucose deprivation disrupts NADP^+^/NADPH balance and elevates ROS production in cancer cells, disturbing cellular redox balance and heightening their susceptibility to cell death [23]. Under glucose deprivation, IDH1 catalyzes the donation of hydrogen ions to NADP^+^, culminating in the production of NADPH [4, 24]. This enzymatic process is crucial for tumor growth and the maintenance of redox balance under low-glucose conditions. In pancreatic cancer, exposure to low glucose levels induces an upregulation in IDH1 expression, potentially linked to stabilization by the RNA-binding protein HuR [25]. The mechanisms governing low glucose-induced IDH1 expression in glioma cells remain unclear [26]. Our research indicates that glucose deprivation results in an increase in IDH1 in CRC, which is at least partially mediated by RIOK3. RIOK3 can enhance the binding between HSP90α and IDH1, thereby promoting IDH1 stability and expression levels.

Over 400 HSP90-dependent substrates, with a substantial proportion playing pivotal roles in fundamental biological processes, have been documented [27]. A few studies have investigated the intricate role of HSP90 in regulating redox homeostasis in CRC. Treatment with HSP90 inhibitors 17-AAG [28] and SNX-2112 [29] has independently been associated with increased ROS levels. Our findings align with these reports, consistently indicating that HSP90α inhibition leads to an increase in ROS levels. Under glucose starvation, GLCC1 stabilizes c-Myc by binding to HSP90, thereby influencing the glucose metabolism of CRC [30]. Our findings further elucidate the pivotal role of HSP90 in mediating the regulatory influence of RIOK3 on IDH1 expression.

However, there are currently no FDA-approved HSP90 inhibitors for clinical use owing to concerns such as cytotoxicity [31]. The development of more selective small-molecule inhibitors may be an effective approach to reduce the side effects of drugs [32]. In subsequent research, we intend to delve deeper into the specific domains responsible for the interaction between RIOK3 and HSP90α and design small-molecule compounds based on their structure to inhibit the interaction between these two components. Given that RIOK3 is an atypical protein kinase, we also aim to investigate whether RIOK3 influences the HSP90α phosphorylation levels and further explore whether this modification subsequently affects its binding capacity with IDH1. We believe that further research in this direction can help provide novel insights into the treatment of CRC.

## Conclusions

In summary, our findings elucidate the pivotal role of RIOK3 in the cellular response to glucose deprivation, demonstrating its capacity to enhance IDH1 expression and NADPH generation in an HSP90α-dependent manner. The identified interaction between RIOK3–HSP90α provides valuable insights into the complex regulatory networks governing CRC survival under metabolic stress. Overall, our findings advance the understanding of CRC adaptation to nutrient challenges, offering novel insights for the development of targeted interventions.

## Materials and methods

### Cell culture

NCM460 (#iCell-h373), HCT116 (#iCell-h071), and SW480 (#iCell-h204) were purchased from iCell Bioscience Inc. (Shanghai, China). FHC (#CTCC-001-0208) was purchased from Zhejiang Meisen Cell Technology Co., Ltd. All cell lines have undergone STR identification within the last six months. Cells were grown in DMEM with 10% (vol/vol) fetal bovine serum and the appropriate amount of penicillin/streptomycin in a 37 °C incubator with a humidified, 5% CO_2_ atmosphere. Mycoplasma contamination was assessed using the universal mycoplasma detection kit (Beyotime Biotechnology, #C0297S) at bi-monthly intervals.

### Plasmid constructions and mutagenesis

The plasmids flag-RIOK3, flag-IDH1, and HA-ubiquitin were purchased from Youbao Bio (Shanghai, China).

The plasmids used for CRISPR-Cas9 gene editing were pSpCas9(BB)-2A-Puro (PX459, Addgene ID 48139). The sequences of the sgRNA oligos used for gene targeting were: RIOK3-1: 5’-GCTCT GAAGA TGAGG TTGAC-3’; RIOK3-2: 5’-GCTGG CTCAG ATGCT ACAGA-3’.

shRNA sequences as used in this study are as follows: shHSP90α: 5’-GTTAT CCTAC ACCTG AAAGA A-3; shIDH1: 5’-CCTAT CATCA TAGGT CGTCA T-3’. All shRNA oligonucleotides were purchased from Shanghai GenePharma Company.

These plasmids were transfected into cells using the Lipofectamine 2000 transfection kit (Invitrogen) according to the manufacturer’s instructions.

### RNA extraction and quantitative real-time PCR

Total RNA was extracted from two cell lines using TRIzol (Invitrogen) according to the manufacturer’s protocol. Random primers were used for reverse transcription. Quantitative PCR was performed using SYBR-Green mix (Invitrogen) and run in a 7500 Real-Time PCR System (Applied Biosystems) with the following cycling parameters: 94 °C for 2 min; 40 cycles of 94 °C for 15 s, 56 °C for 20 s and 72 °C for 30 s; and 72 °C for 2 min. With a melting curve analysis, ΔΔCT analysis was applied to determine qPCR results. Each analysis was performed in triplicate. The qPCR primer sequences are:

β-actin: CCAACCGCGAGAAGATGA, CCAGAGGCGTACAGGGATAG;

RIOK3: AATATGATGCACAGCTTAGGCG, ATCACGAGTATCCTGCCAGTC;

HSP90α: AGGAGGTTGAGACGTTCGC, AGAGTTCGATCTTGTTTGTTCGG;

IDH1: TGTGGTAGAGATGCAAGGAGA, TTGGTGACTTGGTCGTTGGTG.

### Antibodies and reagents

The antibodies used were anti-β-actin (Santa Cruz, #sc-7210), anti-flag-tag (Sigma-Aldrich, #F1804), anti-RIOK3 (abclonal, #A15744), anti-HSP90α (Abcam, #ab282108), anti-IDH1 (proteintech, # 12332-1-AP), and anti-HA-tag (zsbio, #TA-04).

N-Acetyl-L-cysteine (NAC, #ST1546) was purchased from Beyotime Biotechnology. 17-AAG (#S1141) and MG132 (#S2619) were purchased from Selleck. Cycloheximide (CHX, #HY-12320) was purchased from MCE.

### Western blotting

Equal amounts of proteins (20–50 μg) were size fractionated by 6–15% SDS polyacrylamide gel electrophoresis.

### Co-immunoprecipitation (CO-IP)

Cells were harvested and then lysed in lysis buffer (0.5% NP-40, 150 mM NaCl, 50 mM Tris at pH 7.5, 5 mM EDTA, and 1% EDTA-free protease and phosphatase inhibitor cocktails (Roche Applied Science) on ice for 25 min. After centrifugation at 4 °C at 12,000 rpm for 15 min, 2 μg of the indicated antibody was added into the supernatant and incubated at 4 °C overnight. Then, 30 μl of Protein A + G Agarose (Beyotime Biotechnology) was added and incubated for 2 h at 4 °C. The beads were washed with NP-40 buffer three times. The precipitated components were analyzed by western blotting.

### Cell viability

Changed cell viability was assayed using a cell counting kit-8 (CCK-8) kit (Beyotime Biotechnology). In brief, HCT116 and SW480 cells were grown in 96-well plates at a density of 2 × 10^3^ cells/well. At the end of each experiment, cell culture was added with the CCK-8 reagent, and cells were further cultured for 1 h and the optical density of cell culture was then measured at 450 nm. Cell viability was determined in terms of the proportion of cell survival, compared with control.

### Cell death analysis

Cells were seeded into 6-well plates. After different treatments, the medium in the wells and trypsinized cells were collected, spun down, and washed with PBS. Cells were resuspended in 7-aminoactinomycin D (7-AAD) (BD, #559925) solution in PBS and analyzed by flow cytometry.

### Colony formation assay

Cells were plated at the indicated densities onto 6-well plates. 24 h after plating, cells were washed twice with PBS and incubated in high- or low-glucose DMEM for 48 h, which was followed by a recovery period in normal growth medium. After the recovery period, cells were washed with PBS, fixed in methanol: acetic acid (3:1 v/v), and stained using 0.4% crystal violet. The colony area was determined by using the Colony Area plugin and ImageJ software.

### Nude mice tumor xenograft

All mice used in this study were supplied by and housed under specific-pathogen-free conditions at Capital Medical University (Beijing, China). Six-week-old male BALB/c nude mice were used for tumorigenesis and the mice were randomly divided into 4 groups (*n* = 4). 5 × 10^6^ treated HCT116 cells were injected subcutaneously into mice. Treatment commenced when the tumor volume reached 200 mm^3^. 17-AAG was administered daily via intraperitoneal injection at a dosage of 80 mg/kg in a solution comprising 10% DMSO and 90% corn oil. 6 weeks after inoculation, the mice were killed, the tumors were weighed, and the volume was measured. All experiments were performed according to Animal Utilization Protocols and reviewed by the Animal Experiments and Experimental Animal Welfare Committee of Capital Medical University.

### Detection of intracellular ROS levels

ROS levels were determined by incubating the cells with 5 μM dichlorodihydrofluorescein diacetate (DCFDA, KeyGEN BioTECH) for 40 min at 37 °C. Then trypsinized the cells and measured fluorescence using flow cytometry.

### Measurement of NADP^+^/NADPH ratio

The measurement of the NADPH/NADP^+^ ratio was determined using a NADP^+^/NADPH Assay Kit with WST-8 (S0179, Beyotime Biotechnology) according to the manufacturer’s instructions. In brief, 1 × 10^6^ cells cultured in DMEM were collected and lysed by 3 frozen-thaw cycles. Lysate sample was then separated into two portions. One portion was heated at 60 °C to deplete NADP^+^ while the other portion was left on ice as unheated sample (containing both NADP^+^ and NADPH). NADP^+^ could be reduced into NADPH in the working buffer and the NADPH formed further reduced WST-8 to formazan. The orange product (formazan) was then measured at 450 nm spectrophotometrically. The results were normalized by protein concentration of each sample.

### Glucose concentration measurement

Glucose concentration in mice tumors was measured using glucose content assay kit (Solarbio, #BC2505) according to the instructions.

### Immunohistochemical (IHC) staining

For IHC analysis, 3 mm sample sections were incubated with anti-RIOK3 (1:100), and anti-IDH1 (1:100) respectively, overnight at 4 °C in a humidified chamber, followed by incubation with the HRP-conjugated secondary antibodies for 2 h. Staining was completed by 5–10 min incubation with diaminobenzidine (DAB) substrate, which resulted in a brown-colored precipitate at the antigen site.

### Statistical analysis

Data are shown as the mean ± SD for the experiments repeated with at least 3 replicates. The sample size was generally chosen based on preliminary data indicating the variance within each group and the differences between groups. All statistical analyses were performed using GraphPad Prism 9.4.1. Significance of the difference between two groups was determined using unpaired Student’s t-tests after accounting for equality of variances using an F test. n.s. *p* > 0.05, *p < 0.05, **p < 0.01.

### Supplementary information

Supplemental Figures

## Data Availability

All the raw data of this study are available from the corresponding author upon reasonable.
