# Supplementary material for: RIOK3 sustains colorectal cancer cell survival under glucose deprivation via an HSP90α-dependent pathway
Source: Oncogenesis. 2024 Mar 7;13(1):12. doi: 10.1038/s41389-024-00514-5 (PMC10920805; doi:10.1038/s41389-024-00514-5)
Supplement: Supplementary file 1 — Supplemental Figures [file 41389_2024_514_MOESM1_ESM.docx]

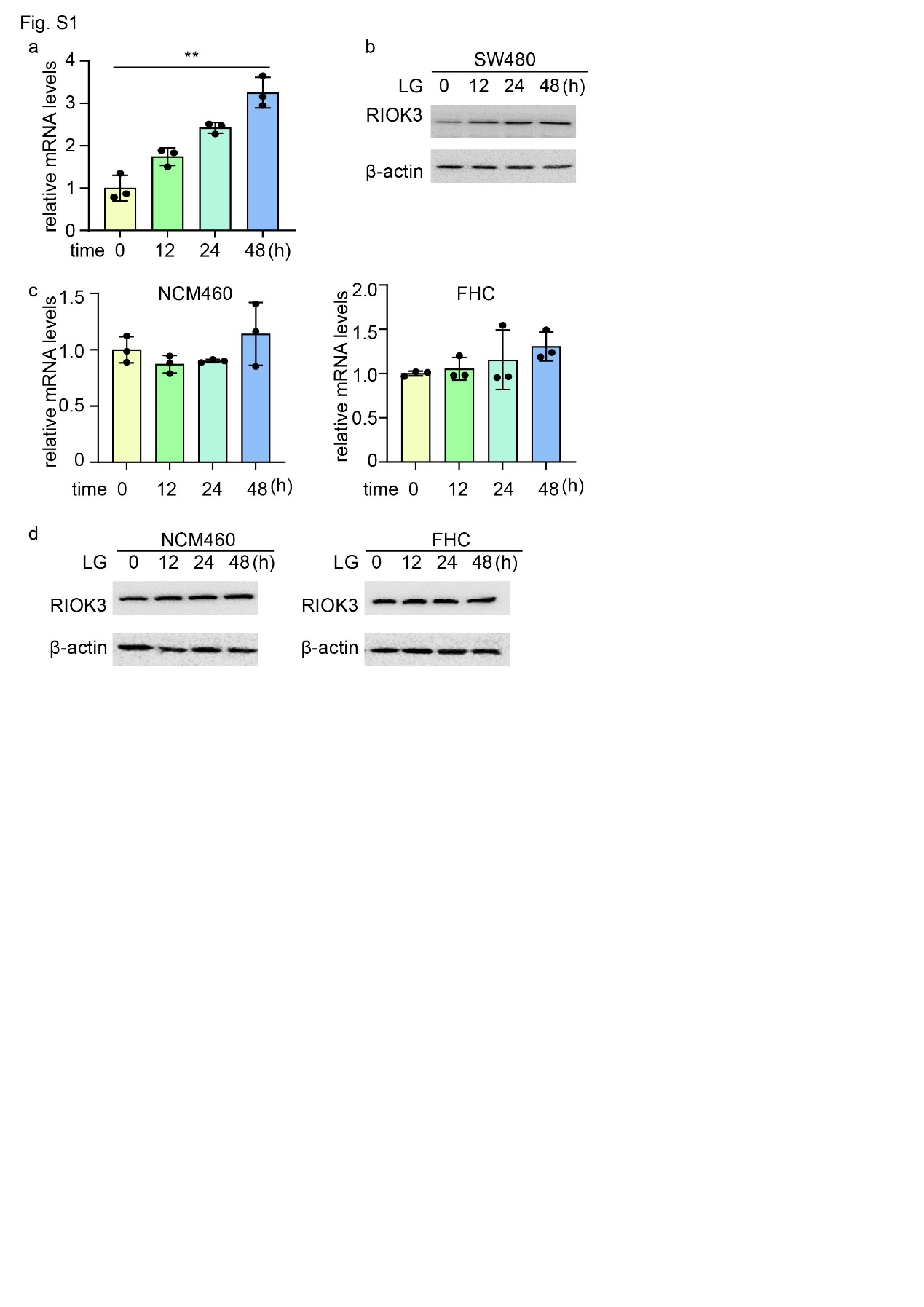


## Fig. S1 The impact of glucose depletion on RIOK3 expression in colon cells. Related to Fig. 1

a. SW480 cells were incubated in low-glucose DMEM for the indicated times. RNA was then extracted and RIOK3 mRNA levels were analyzed with qPCR. Data are shown as mean ± SD (n=3).

b. SW480 cells were incubated in low-glucose DMEM for the indicated times. Western blotting was performed to detect RIOK3 protein levels.

c. NCM480 and FHC cells were incubated in low-glucose DMEM for the indicated times. RNA was then extracted and RIOK3 mRNA levels were analyzed with qPCR. Data are shown as mean ± SD (n=3).

d. NCM480 and FHC cells were incubated in low-glucose DMEM for the indicated times. Western blotting was performed to detect RIOK3 protein levels.


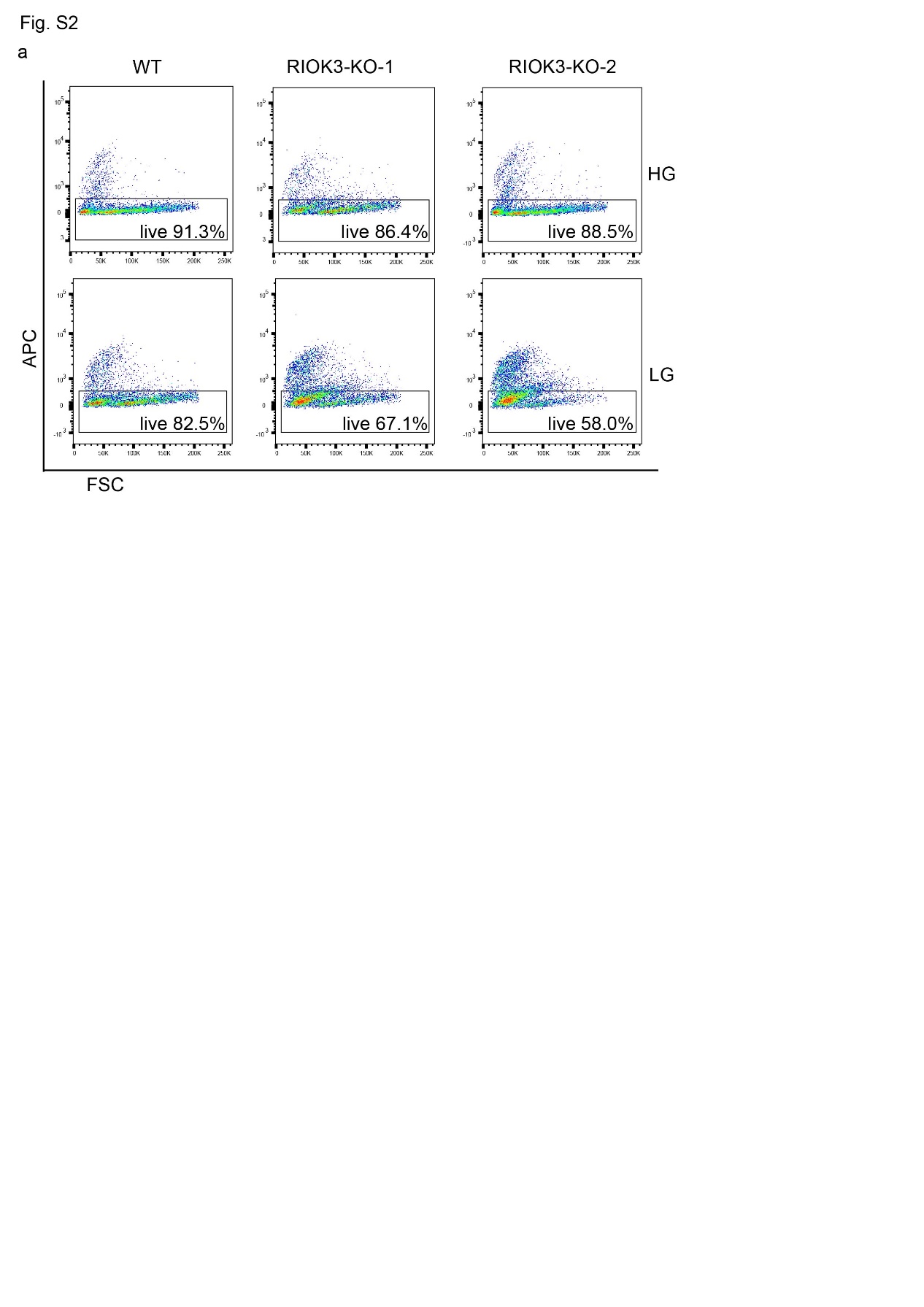


## Fig. S2 RIOK3 is essential for the survival of CRC under glucose starvation. Related to Fig. 2

a. WT and RIOK3-KO HCT116 cells were cultured in low-glucose DMEM for 48 hours, and the cell death rate was measured by 7-AAD staining assay. The representative flow cytometry results were presented.


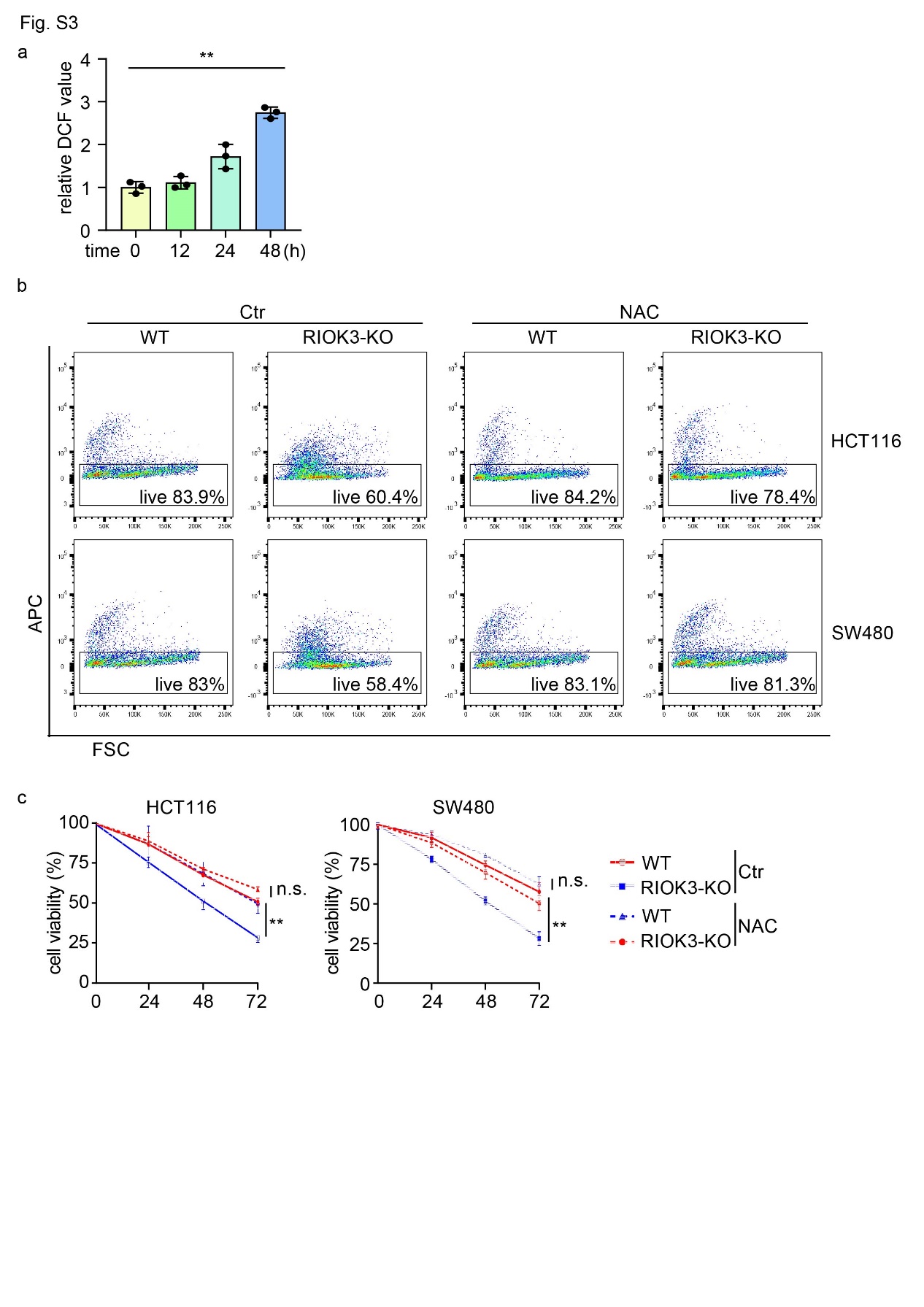


## Fig. S3 NAC reversed the high cell death rate of RIOK3-KO cells. Related to Fig. 3

a. HCT116 cells were incubated in low-glucose DMEM for the indicated times, and the ROS levels were quantified as the mean DCF values. Data are shown as mean ± SD (n=3).

b. WT and RIOK3-KO CRC cells were cultured in low-glucose DMEM in the presence or absence of 10 mM NAC and the cell death rate was measured by 7-AAD staining assay. The representative flow cytometry results were presented.

c. WT and RIOK3-KO CRC cells were cultured in low-glucose DMEM in the presence or absence of 10 mM NAC. Cell viability was then tested with the CCK-8 assay. Data are shown as mean ± SD (n=3).


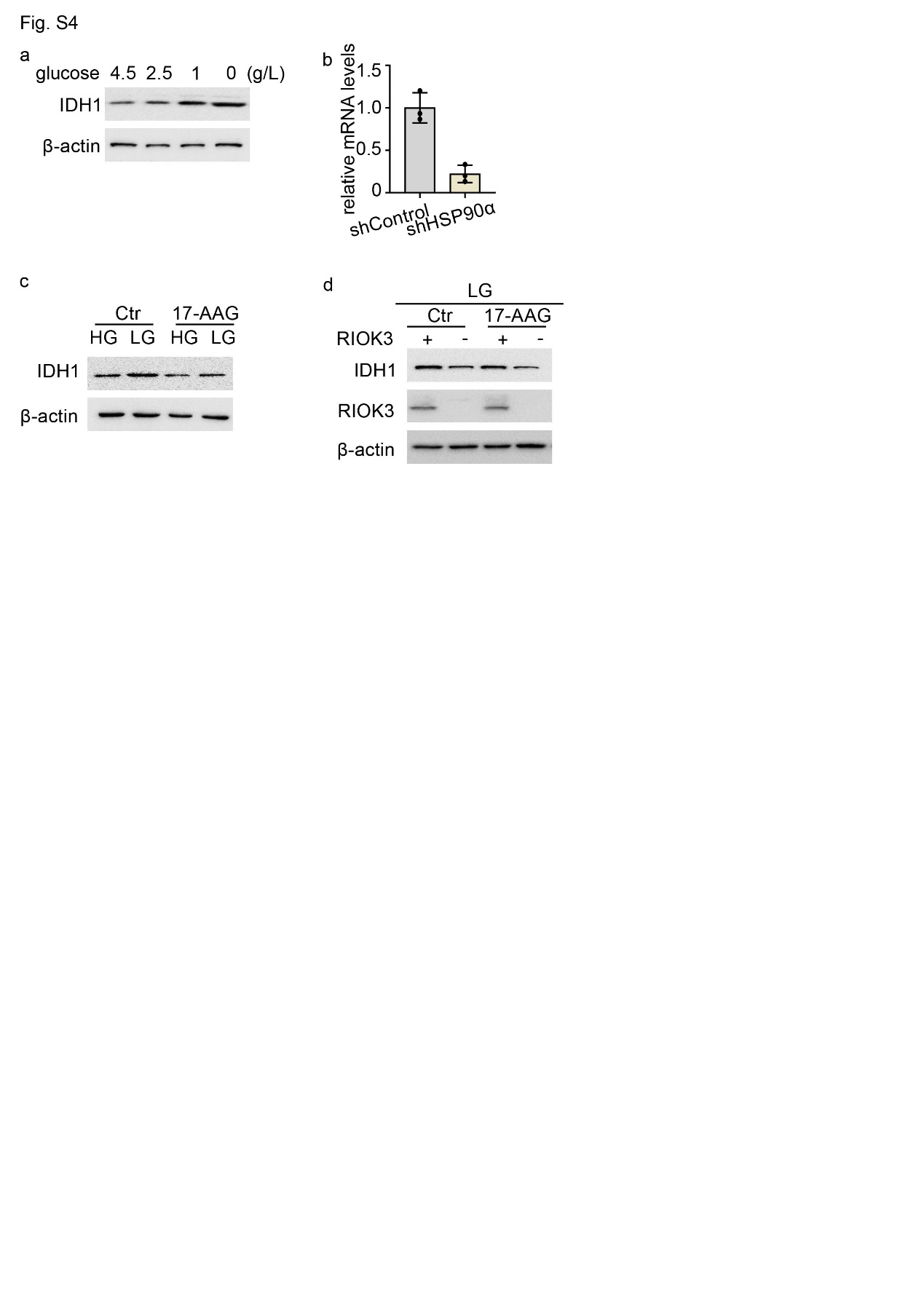


## Fig. S4 HSP90 inhibition modulates IDH1 expression. Related to Fig. 4

a. HCT116 cells were cultured in a medium with a specific glucose concentration for 24 hours. Western blotting was performed to detect IDH1 protein levels.

b. RNA was extracted and HSP90α mRNA levels were analyzed with qPCR. Data are shown as mean ± SD (n=3).

c. HCT116 cells were cultured in low-glucose DMEM with 400 nM 17-AAG for 48 hours. Western blotting was performed to detect IDH1 protein levels.

d. WT and RIOK3-KO HCT116 cells were cultured in low-glucose DMEM with 400 nM 17-AAG for 48 hours. Western blotting was performed to detect IDH1 protein levels.


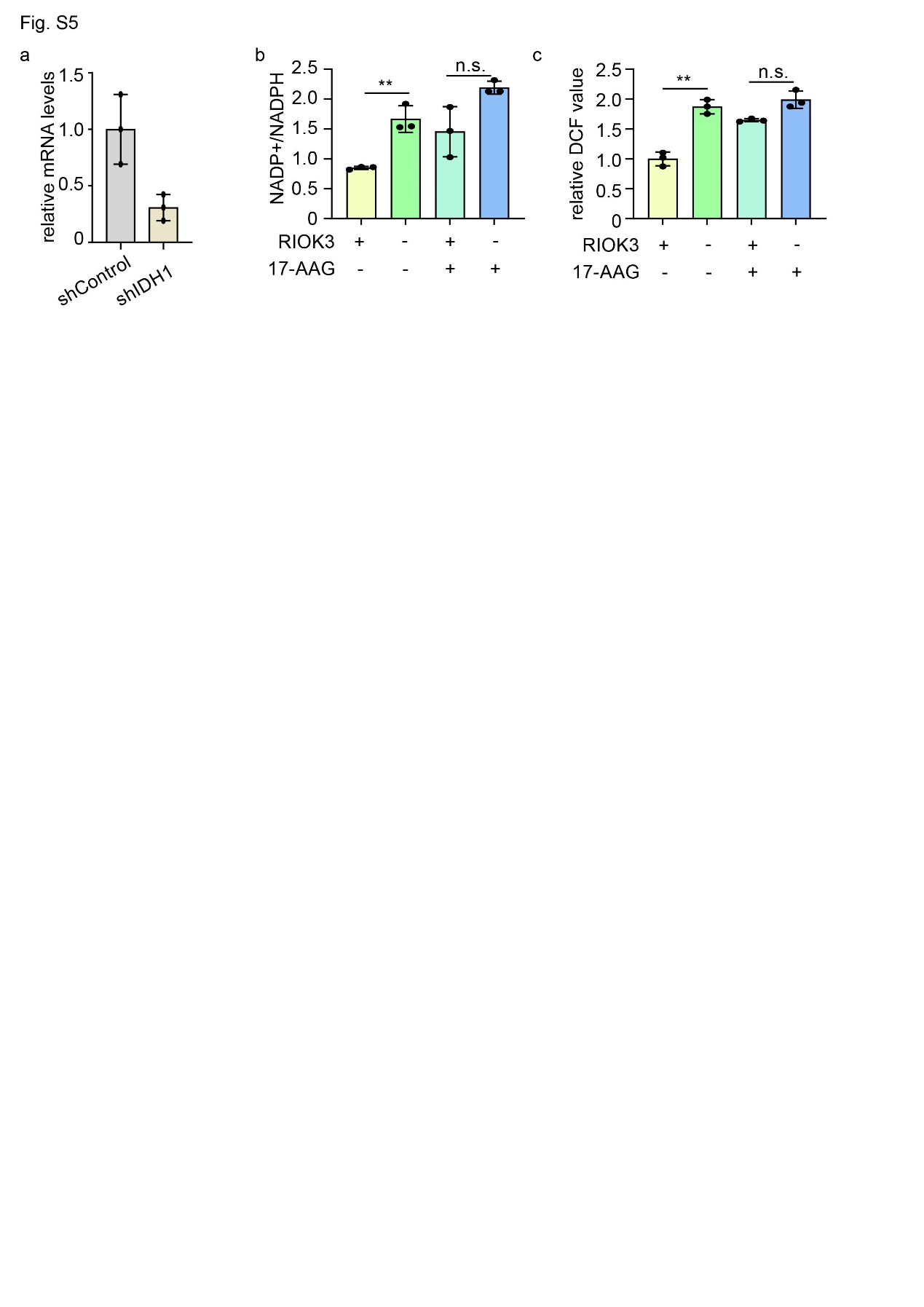


## Fig. S5 RIOK3 maintains redox balance through HSP90α. Related to Fig. 5

a. RNA was extracted and IDH1 mRNA levels were analyzed with qPCR. Data are shown as mean ± SD (n=3).

b. WT and RIOK3-KO HCT116 cells were cultured in low-glucose DMEM with 400 nM 17-AAG for 48 hours. The NADP^+^/NADPH ratio was then determined. Data are shown as mean ± SD (n=3).

c. WT and RIOK3-KO HCT116 cells were cultured in low-glucose DMEM with 400 nM 17-AAG for 48 hours. ROS levels were then quantified as the mean DCF values. Data are shown as mean ± SD (n=3).

##
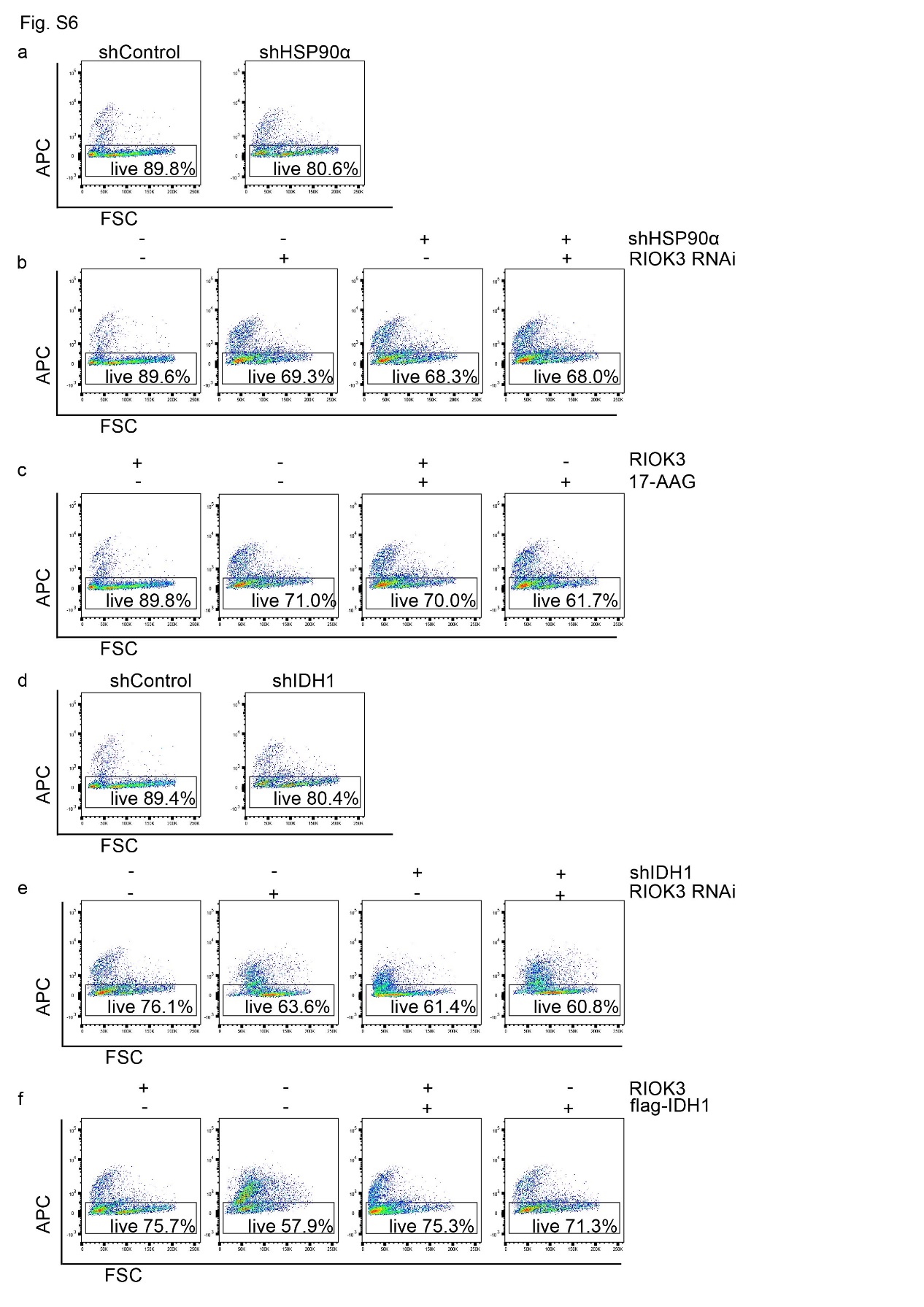
Fig. S6 RIOK3 maintains CRC cell survival through HSP90α. Related to Fig. 6

a. HCT116 cells expressing control-shRNA or HSP90α-shRNA were cultured in low-glucose DMEM for 48 hours, and the cell death rate was measured by 7-AAD staining assay. The representative flow cytometry results were presented.

b. HCT116 cells expressing control-shRNA or HSP90α-shRNA were transfected with a non-specific siRNA or RIOK3 siRNA. 24 hours after transfection, cells were cultured in low-glucose DMEM for 48 hours, and the cell death rate was measured by 7-AAD staining assay. The representative flow cytometry results were presented.

c. WT and RIOK3-KO HCT116 cells were cultured in low-glucose DMEM with 400 nM 17-AAG for 48 hours, and the cell death rate was measured by 7-AAD staining assay. The representative flow cytometry results were presented.

d. HCT116 cells expressing control-shRNA or IDH1-shRNA were cultured in low-glucose DMEM for 48 hours, and the cell death rate was measured by 7-AAD staining assay. The representative flow cytometry results were presented.

e. HCT116 cells expressing control-shRNA or IDH1-shRNA were transfected with a non-specific siRNA or RIOK3 siRNA. 24 hours after transfection, cells were cultured in low-glucose DMEM for 48 hours, and the cell death rate was measured by 7-AAD staining assay. The representative flow cytometry results were presented.

f. WT and RIOK3-KO HCT116 cells were transfected with an empty vector or flag-IDH1 plasmids, 24 hours after transfection, cells were cultured in low-glucose DMEM for 48 hours, and the cell death rate was measured by 7-AAD staining assay. The representative flow cytometry results were presented.


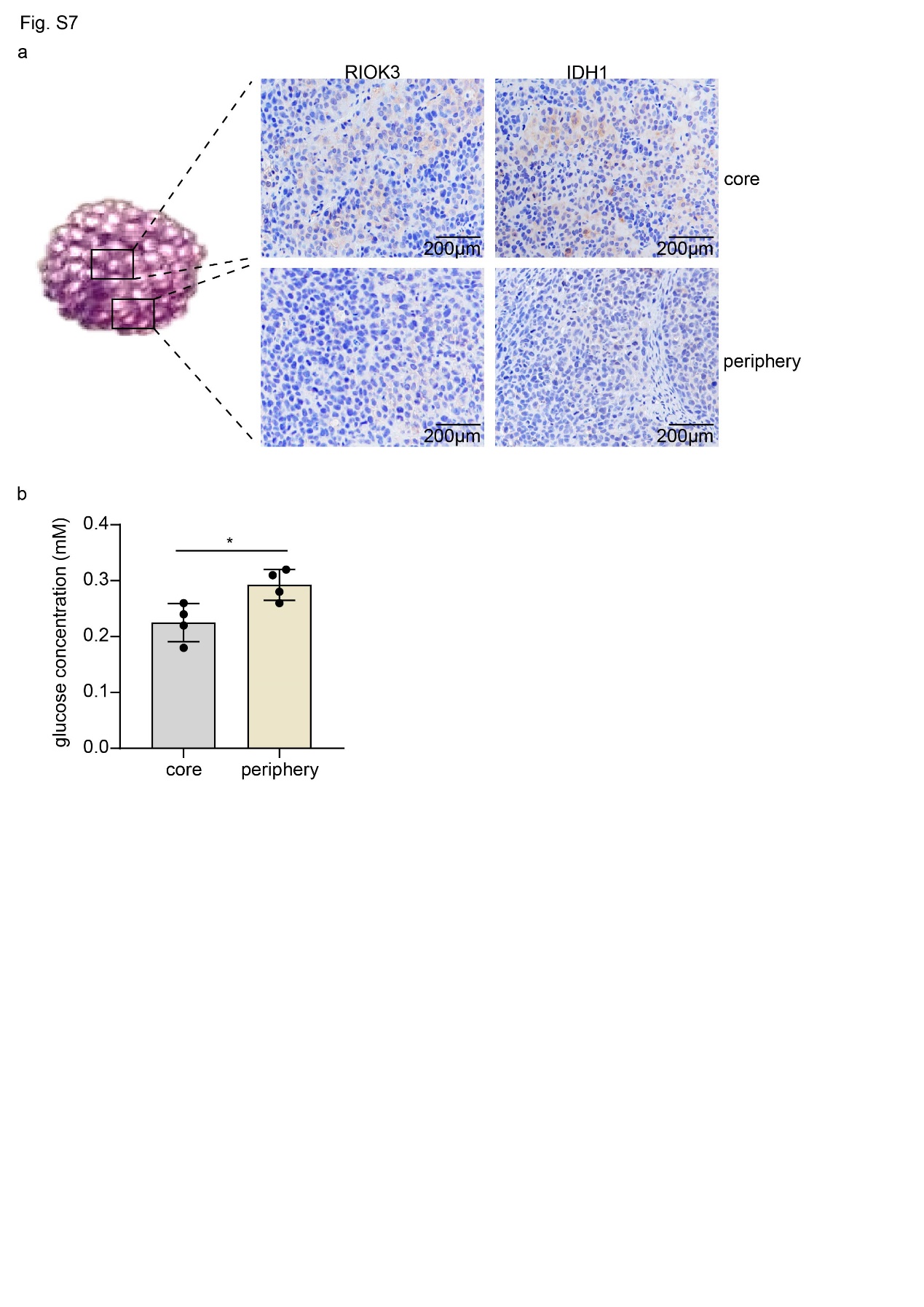


## Fig. S7 RIOK3 assists CRC cells in surviving under glucose deprivation. Related to Fig. 6

a. Immunohistochemical staining of RIOK3 and IDH1 in mice xenograft.

b. Glucose concentrations of the tumors (n=4).
